# Optimizing *Agrobacterium*-Mediated Transformation and CRISPR-Cas9 Gene Editing in the *tropical japonica* Rice Variety Presidio

**DOI:** 10.3390/ijms222010909

**Published:** 2021-10-09

**Authors:** Marco Molina-Risco, Oneida Ibarra, Mayra Faion-Molina, Backki Kim, Endang M. Septiningsih, Michael J. Thomson

**Affiliations:** 1 Department of Soil and Crop Sciences, Texas A&M University, College Station, TX 77843, USA; marco.molinarisco@ag.tamu.edu (M.M.-R.); oibarra6387@gmail.com (O.I.); mayra.molina@ag.tamu.edu (M.F.-M.); uptfamily@snu.ac.kr (B.K.); eseptiningsih@tamu.edu (E.M.S.); 2 Avance Biosciences Inc., Houston, TX 77040, USA; 3 Plant Genomics and Breeding Institute, Seoul National University, Seoul 08826, Korea

**Keywords:** rice (*Oryza sativa* L.), *tropical japonica*, gene editing, CRISPR/Cas9, *phytoene desaturase* (*PDS*)

## Abstract

Bottlenecks in plant transformation and regeneration have slowed progress in applying CRISPR/Cas-based genome editing for crop improvement. Rice (*Oryza sativa* L.) has highly efficient *temperate japonica* transformation protocols, along with reasonably efficient *indica* protocols using immature embryos. However, rapid and efficient protocols are not available for transformation and regeneration in *tropical japonica* varieties, even though they represent the majority of rice production in the U.S. and South America. The current study has optimized a protocol using callus induction from mature seeds with both *Agrobacterium*-mediated and biolistic transformation of the high-yielding U.S. *tropical japonica* cultivar Presidio. Gene editing efficiency was tested by evaluating knockout mutations in the *phytoene desaturase* (*PDS*) and *young seedling albino* (*YSA*) genes, which provide a visible phenotype at the seedling stage for successful knockouts. Using the optimized protocol, transformation of 648 explants with particle bombardment and 532 explants with *Agrobacterium* led to a 33% regeneration efficiency. The *YSA* targets had ambiguous phenotypes, but 60% of regenerated plants for *PDS* showed an albino phenotype. Sanger sequencing of edited progeny showed a number of insertions, deletions, and substitutions at the gRNA target sites. These results pave the way for more efficient gene editing of *tropical japonica* rice varieties.

## 1. Introduction

Recent advances in CRISPR/Cas-based gene editing have led to a renewed interest in optimizing plant transformation and regeneration techniques to enable more rapid development of gene edited lines for crop improvement. *Agrobacterium tumefaciens*-mediated transformation and biolistic/particle bombardment-based delivery have been used for plant transformation for over 30 years [1,2], with advantages and disadvantages to each method. Transformation using binary vectors for *Agrobacterium*-mediated delivery and integration of the T-DNA often results in single copy insertions, but has species and genotype-specific differences in protocols and efficiencies. At the same time, biolistic-based delivery has fewer constraints on the target species, but often results in multiple insertions or insertion of partial DNA fragments. Both delivery methods require slow, labor-intensive, and often complex sets of species- and genotype-specific protocols for in vitro tissue culture and regeneration from transformed explants. 

The past few decades have seen significant improvements in tissue culture techniques which have drastically increased the number of species capable of successfully passing through the lengthy tissue culture process. In addition to the components of the culture media, the explant origin has long been known to play an important role in tissue culture success [3]. Even problems in otherwise simple steps such as embryogenic calli formation can be difficult to overcome, especially in recalcitrant crops. Often, the plant’s regeneration step is a greater bottleneck than the gene integration itself. Most crop gene editing studies to date have employed model genotypes that are relatively easy to transform; however, gene editing will need to be performed in high-yielding modern cultivars to make an immediate impact in plant breeding. Unfortunately, for many crops, modern cultivars and elite breeding lines, unlike the model genotypes, tend to be tissue culture recalcitrant, and attempting to directly apply novel gene editing techniques to manipulate desirable agronomic traits in high-performing breeding lines is not an easy task. Thus, although in vitro plant transformation, tissue culture, and regeneration techniques have made tremendous progress over the past few decades, there are still several bottlenecks that hamper faster progress, including low frequency of transformed events, long periods of time needed for tissue culture and regeneration, and low precision of biolistic-mediated transformation [4]. Therefore, to benefit from the advantages of gene editing technology, callus induction, *Agrobacterium*-mediated transformation (preferred for its precision and single copy insertions), and plant regeneration must be optimized for routine transformation and delivery of CRISPR reagents.

As a model monocot species, rice (*Oryza sativa*) has a long history of research to optimize *Agrobacterium*-mediated transformation and regeneration; however, this has largely focused on relatively easy-to-transform *temperate japonica* varieties such as Nipponbare and Kitaake [5,6,7,8,9,10]. These varieties have efficient callus induction from seeds, high efficiency of transformation by *Agrobacterium*, and good regeneration capacity from callus tissue [11]. However, previous research has indicated that it is much more difficult to achieve successful transformation and tissue culture in other rice subgroups. For example, an early study examined rates of callus induction and regeneration across 60 rice genotypes using mature seeds and found much lower rates of callus induction and regeneration in *indica* and *tropical japonica* (previously referred to as “*javanica*”), in comparison to *temperate japonica* [12]. For recalcitrant rice genotypes that have difficulty in callus induction from mature seeds, greater success has been made starting from immature embryos, especially for *indica* varieties, although with the added downside of having to grow plants to the flowering stage and harvest and extract the embryos from the immature seeds [9,13,14,15,16,17,18,19,20]. 

In contrast with the large number of studies working to improve *indica* transformation efficiency, just a handful of studies have demonstrated successful transformation of *tropical japonica* rice varieties. One early study showed a significantly lower transformation efficiency for the *tropical japonica* variety “Lemont” in comparison to the *indica* “IR64” using seedling shoots as initial explants [21]. Subsequently, *Agrobacterium*-mediated transformation using immature embryos was optimized for two U.S. *tropical japonica* varieties, and transformation efficiencies ranged from 4–15% for Jefferson and 9–20% for Gulfmont [22]. Another study optimized the transformation frequency of the Indonesian *tropical japonica* variety “Rojolele” to 23% of the efficiency of Nipponbare [23]; while the same group found a lower transformation efficiency for four other *tropical japonica* varieties, ranging from 3.75 to 10% using callus induction from mature seeds [24]. A more recent study optimized transformation and regeneration for the Indonesian *tropical japonica* black rice variety “Cempo Ireng,” but ended up with a transformation efficiency of 1.5% using callus induction from mature seeds [25]. These studies suggest that many *tropical japonica* genotypes are even more difficult to transform and regenerate than *indica* varieties. 

Phytoene desaturase (PDS) is a key enzyme in the carotenoid biosynthesis pathway and plays a critical role in the production of carotenoids, xanthophylls, alpha-carotene, and beta-carotene [26]. Knocking out the function of the *PDS* gene leads to albinism and slow plant growth [27,28]. As such, *PDS* has been used as an easy visual phenotypic marker for CRISPR/Cas9-mediated gene knockouts in various plant species, including rice, tobacco, apple, tomato, cassava, banana, wheat, and melon [29,30,31,32,33,34,35,36,37]. Another gene that may provide a visual phenotypic marker for gene knockouts in rice is the *young seedling albino* (*YSA*) mutant, which affects the levels of total chlorophyll content in young rice seedlings [38]. YSA is a member of a large family of pentatricopeptide repeat (PPR) proteins and plays a role in chloroplast development. A gamma-ray induced *YSA* mutant was characterized as having an albino phenotype before the three-leaf stage, but then turned green again by the six-leaf stage after recovering all pigment levels, including total chlorophyll and carotenoids [38]. Subsequently, knocking out the *YSA* gene in rice with CRISPR/Cas9 was confirmed to result in a young seedling albino phenotype [39].

Although the majority of global rice production is *indica* rice, most rice varieties grown in the Southern U.S. and in South America are *tropical japonica*. For example, Presidio, a long-grain *tropical japonica* cultivar, is a high-yielding rice variety with excellent milling quality, strong resistance to blast and sheath blight diseases, and good ratooning potential for rice-growing areas in Texas and Louisiana [40]. Even though it was released over 15 years ago, it is still the second most popular inbred long-grain cultivar grown in Texas (after the Clearfield variety CL153) and has been grown in Texas on average over 9000 hectares per year the past four years [41]. Unfortunately, transformation of Presidio using the established Nipponbare protocol has been unsuccessful in our hands, indicating that this cultivar needs optimization for tissue culture and regeneration (unpublished data). In this study, we report the optimization of the rice transformation and regeneration protocol of Presidio with mature seeds and confirmation of efficient CRISPR/Cas9 gene editing by creating knockout mutants of *phytoene desaturase* (*PDS*) and *young seedling albino* (*YSA*) using both *Agrobacterium*-mediated transformation and particle bombardment. Optimization of an efficient mature-seed transformation protocol for this *tropical japonica* variety provides rapid and efficient CRISPR-based gene editing in the background of a popular, high yielding, long-grain rice cultivar, paving the way for more efficient gene editing across similar *tropical japonica* varieties for rice production in the U.S. and South America.

## 2. Results

### 2.1. Design and Validation of Guide RNAs for PDS and YSA in Presidio

Primers amplifying several initial exons of the PDS and YSA target genes were designed using the cultivar Nipponbare *Oryza sativa* ssp. *japonica* reference genome [42]. Amplicons obtained from Presidio were sequenced and compared with the Nipponbare reference genome: sequenced regions showing identical sequences were then used to design two guide RNAs (gRNAs) for each gene target. One gRNA targeting the PDS gene was designed to exon 3, while a second gRNA was designed to exon 4; both gRNAs targeting the YSA gene were designed to the first exon (Supplementary Table S1). 

**Figure 1:**
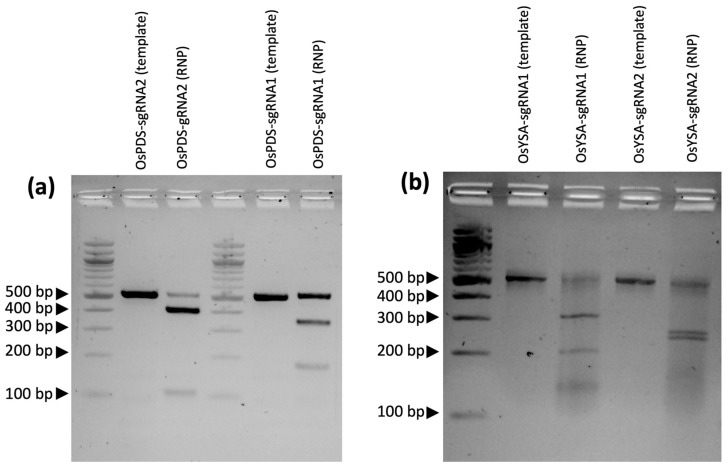
Gel electrophoresis showing in vitro ribonucleoprotein (RNP) assay results with digestion of PCR amplicons using commercial Cas9 with (**a**) PDS gRNAs and (**b**) YSA gRNAs. The gel images show a DNA ladder in lane 1, followed by two reactions for each gRNA: one with the template only, which lacks the gRNA and is uncut, and the second with the functional RNP containing the gRNA showing the PCR amplicons cut into two products, as follows (from left to right): OsPDS-sgRNA2: 515 bp product cleaved into 409 bp and 106 bp fragments; OsPDS-sgRNA1: 515 bp product cleaved into 333 bp and 182 bp fragments; OsYSA-sgRNA1: 526 bp product cleaved into 321 bp and 205 bp fragments; OsYSA-sgRNA2: 526 bp product cleaved into 268 bp and 258 bp fragments.

To validate that the gRNAs function properly, synthetic gRNAs were ordered and tested using an in vitro ribonucleoprotein (RNP) assay with commercial Cas9 enzyme to cut PCR amplicons flanking the target sites. The two PDS gRNAs cleaved the 515 bp amplicon into two fragments each: OsPDS-sgRNA1 generated the expected fragments of 333 bp and 182 bp, while OsPDS-sgRNA2 generated the expected 409 bp and 106 bp fragments (Figure 1a). Gel image analysis showed that the first PDS gRNA cleaved approximately 45% of the target fragment, and the second gRNA cut 83% of the fragment when compared to the control. Both YSA gRNAs targeting the first exon also generated the expected fragment sizes when combined with Cas9: OsYSA-sgRNA1 generated 321 bp and 205 bp fragments and OsYSA-sgRNA2 produced 268 bp and 258 bp fragments from a 526 bp amplicon (Figure 1b). Gel image analysis revealed that the first YSA gRNA cleaved 67% of the target DNA fragment, and the second sgRNA cleaved 51%. These results showed that all four gRNAs were successful in cleaving the target DNA sites.

### 2.2. Agrobacterium-Mediated Transformation of Presidio

The tissue culture transformation approach using callus induction from mature seeds was optimized starting from a previously published protocol [9] with successful modifications on several major steps, including extended calli induction period, calli selection, lighting condition, plant regeneration length, and media composition (Table 1). 

**Figure 2:**
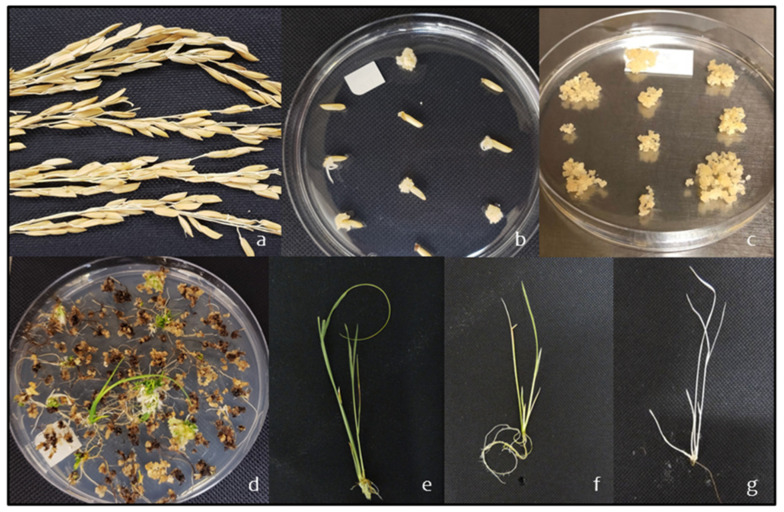
Transformation and regeneration steps for gene editing in the Presidio background. (**a**) Mature seeds were collected from Presidio plants, (**b**) placed on calli induction media, and (**c**) allowed to form calli, which were then cut into pieces and treated with *Agrobacterium tumefaciens* strain EHA105 with the pRGEB32 binary vector containing Cas9 and the targeted gRNAs. (**d**) Transformed calli treated with *Agrobacterium* were placed on regeneration media and allowed to grow. Phenotypes were evaluated at the seedling stage, revealing (**e**) wild type, (**f**) variegated, and (**g**) albino plants.

In brief, well-maintained mature Presidio seeds (Figure 2a) were dehusked, surface sterilized, placed on callus induction medium, and incubated in the dark for 7 days (Figure 2b). Scutella from cultured seeds were then transferred to fresh medium and incubated in the dark for calli formation. Afterward, the calli were then cut into small pieces of 0.5–1 mm in diameter and transferred to fresh medium (Figure 2c). The plates were then incubated under continuous illumination at 32 °C for 3 days. Subsequently, a bacterial suspension of *Agrobacterium tumefaciens* strain EHA105 with the pRGEB32 binary vector containing Cas9 and two sgRNAs for each gene target was mixed with the calli and incubated for 2 min. Approximately 150 calli were transferred with forceps to a co-cultivation medium and incubated in the dark at 25 °C for 1 day. After co-cultivation, the calli were transferred to selective medium and incubated under dark conditions for 2 weeks in the first round of selection, followed by 7 days for a second round on the selection medium. The proliferated, yellowish white calli were transferred to regeneration medium and incubated under continuous illumination. Thereafter, the subculture was performed into fresh media every three weeks until plant regeneration, at which time the seedling phenotypes were evaluated (Figure 2d–g). A total of 211 explants were transformed with *Agrobacterium tumefaciens* containing the pRGEB32-PDS-sgRNAs and 321 explants were transformed with the pRGEB32-YSA-sgRNAs (Table 2). All explants were placed under hygromycin selection and underwent regeneration. Total regeneration efficiency for *Agrobacterium*-mediated transformation across all 532 explants was 27% (Table 2).

### 2.3. Particle Bombardment-Mediated Transformation of Presidio

Presidio rice calli were prepared as above for use in particle bombardment-mediated transformation with the gene gun (Biolistic® PDS-1000/He particle delivery system). An aliquot of the gold microcarriers was prepared and plasmid DNA was added to the gold microcarriers and allowed to air dry on the macrocarrier. Presidio rice calli on osmotic media were bombarded and placed in the dark at 25 °C overnight. After the overnight incubation in the dark, plates were kept under the same conditions as calli treated with *Agrobacterium*. A total of 309 explants were transformed using particle bombardment containing the pRGEB32-PDS-sgRNAs and 339 explants with pRGEB32-YSA-sgRNAs. After growing on selectable media, a 38% regeneration rate was observed across the 648 transformed explants (Table 2).

### 2.4. Successful Gene Editing of the PDS and YSA Gene Targets

For the experiment targeting the *PDS* gene, 30 out of the 49 successfully regenerated *Agrobacterium*-transformed plants presented an albino phenotype, while 34 out of the 57 regenerated biolistic-transformed plants presented an albino phenotype (Table 2). Taken together, these results reveal that 60% of regenerated plants targeting the *PDS* gene showed an albino phenotype, which corresponds to a 12% albino phenotype efficiency from the original 532 explants. Targeting the *PDS* gene produced three types of visible phenotypes compared to the green empty vector control: variegated plants, dwarf albino plants, and normal-size albino plants (Supplementary Figure S1). Meanwhile, the mutations from targeting the *YSA* gene had more subtle phenotypic differences when compared to the green empty-vector control: variegated leaves characterized by both white and green streaks or variegated plants with white leaf tips (Supplementary Figure S2). As similar regeneration and phenotypic results were obtained between the biolistic and *Agrobacterium*-mediated transformants, a subset of the *Agrobacterium*-transformed plants was selected for confirmation by sequencing, as *Agrobacterium*-mediated transformation is preferred for having a higher frequency of single-copy insertions. 

**Figure 3:**
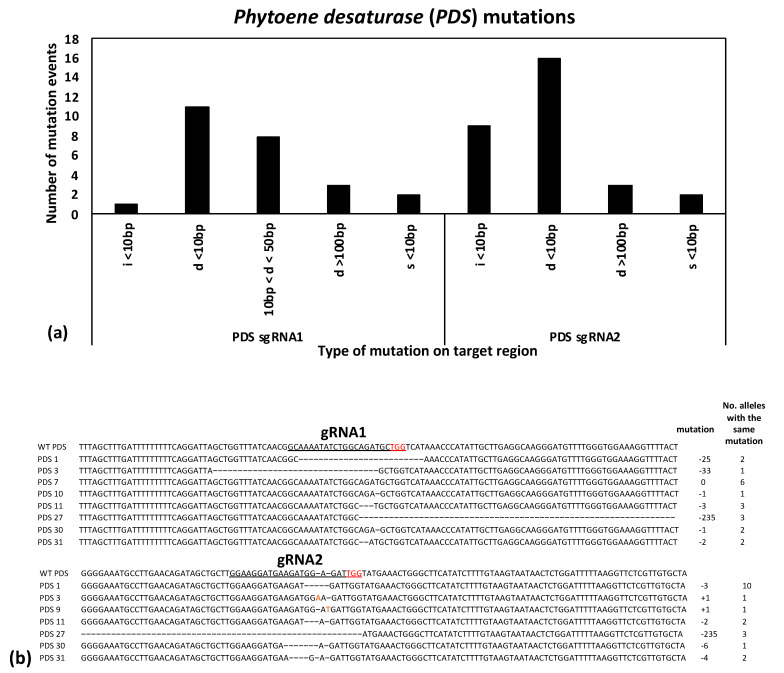
(**a**) Histogram showing the number of different mutation events, including insertions (i), deletions (d), and base substitutions (s), across all edited plants at the *PDS* target site for the two gRNAs; and (**b**) multiple sequence alignments showing mutations detected at the *PDS* target site for gRNA1 and gRNA2, which are 204 base pairs apart. The wild type (WT) *PDS* sequence is shown at top with the gRNA underlined and the PAM underlined and in red, followed by representative samples with differing mutations. The type of mutation is shown as the number of base pairs inserted (+) or deleted (-), followed by the number of alleles with the same mutation detected across the edited progeny. Three alleles were detected with a 235 bp deletion event spanning the *PDS* gRNA1 and gRNA2. The red bases in gRNA2 represent single base insertions.

**Figure 4:**
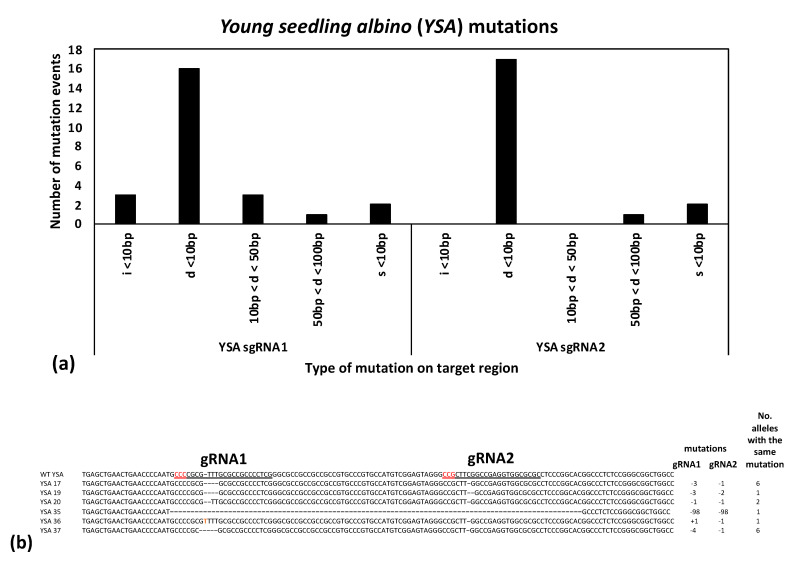
(**a**) Histogram showing the number of different mutation types, including insertions (i), deletions (d), and base substitutions (s), for the *YSA* target site; and (**b**) mutations detected at the *YSA* target site for gRNA1 and gRNA2, which are 40 base pairs apart. The wild type (WT) *YSA* sequence is shown at top, with the two gRNAs underlined and the PAM sites underlined and in red, followed by representative samples with differing mutations. The type of mutation is shows as the number of base pairs inserted (+) or deleted (-), followed by the number of alleles with the same mutation detected across the edited progeny. One allele showed a 98 bp deletion spanning gRNA1 and gRNA2. A single base insertion is shown in red at the gRNA1 site.

A subset of 18 plants from the regenerated *PDS* gRNA transformants was submitted for Sanger sequencing, while 16 of the *YSA* gRNA transformants were sequenced, along with 5 control (wild type) samples (Table 2). PCR products were cloned, and five colonies were sequenced for each sample in order to distinguish biallelic versus monoallelic mutations (Supplementary Figure S3). Sequence analysis revealed a variety of insertions, deletions, and base substitutions detected at both the *PDS* and *YSA* gene target sites. Small deletions (less than 10 bp in length) were the most common mutation type for both *PDS* and *YSA*, followed by < 10 bp insertions, base substitutions, 10–50 bp long deletions, and then deletions greater than 100bp in length for *PDS* (Figure 3a), and 50–100 bp deletions for *YSA* (Figure 4a). For the *PDS* target gene, gRNA2 had more mutations than gRNA1 (Figure 3a), while for *YSA*, gRNA1 had a higher number of mutations than gRNA2 (Figure 4a). As two gRNAs were included in the transformation vectors, many of the edited plants contained mutations at both gRNA sites in the same plant, including one plant for PDS that had a 235 bp deletion event spanning the two gRNA sites (Figure 3b) and one plant for YSA that had a 98 bp deletion event spanning the two gRNA sites (Figure 4b).

## 3. Discussion

The current study has optimized an efficient transformation and gene editing system for the popular U.S. long grain *tropical japonica* variety Presidio. As Presidio could not be transformed using the previous best practice protocol for rice transformation [9], a number of modifications were tested and found necessary to proceed to the next step of the tissue culture process. First, during the callus induction phase, 10 days were not adequate for the induction; only after increasing to 21 days was it possible to achieve actively proliferating calli to be used as initial explants prior to the infection/bombardment step. A similar result was observed by another group working on elite *japonica* varieties [11]. Next, on the *Agrobacterium*-mediated transformation pipeline, co-cultivation can be challenging in an in vitro environment as bacteria overgrowth can lead to irreversible damage to the tissue, jeopardizing further steps. In our hands, the three days of co-cultivation had to be reduced to one day to avoid bacteria overgrowth. Another major change implemented during the calli selection phase was to keep the samples in the dark. During optimization, we noticed that light conditions for Presidio accelerated lethal tissue necrosis on the explants when compared to dark conditions; however, the putative transformed tissue was able to survive and proliferate for both selection stages when kept in the dark. Lastly, hormones play a very important role during the critical regeneration stage and are known to have varying levels of effectiveness across different ranges of concentration, hormone combinations, and length of exposure. After negative regeneration results were obtained using 0.5mg/L kinetin concentration for 14 days as proposed by the literature [9], modifications were implemented to stimulate regeneration responses from the embryogenic tissue. As described in several *indica* rice transformation studies [11,43], often higher concentrations of cytokine and a longer regeneration phase are required. Presidio regeneration was finally successfully obtained using 40 days under 3mg/L of kinetin. Only after identifying the ideal combination of modifications described above were we able to develop a robust transformation protocol for Presidio ready to support a successful gene editing approach.

The first step to ensure a successful gene knockout project is robust gRNA design targeting coding sequences near the start of the gene to increase the chance that a frameshift mutation will lead to a premature stop codon that will knock out the gene function. Best practices call for sequencing the target site in the genotype of interest to detect any polymorphisms that differ from the reference genome that might interfere with the gRNA or PAM sequences. In the current study, Presidio was sequenced at the target regions to avoid any polymorphic regions when designing the gRNAs. Once the target regions were identified, an online gRNA design tool, in this case CRISPRdirect, was used to ensure proper design of the gRNA next to a protospacer adjacent motif (PAM) sequence, while at the same time avoiding potential off-target sites across the genome that may share similar sequences. Another validation step is running the guide RNAs through an in vitro ribonucleoprotein (RNP) assay to ensure that the gRNAs are functional in cutting the target sequence. Interestingly, in the current study, the predicted efficiency of each guide RNA based on how well each gRNA digested the target DNA fragment in the in vitro RNP assays (Figure 1) matched the relative mutation rates from the transformation experiment: for the PDS target gene, gRNA2 had more mutations than gRNA1, while for YSA, gRNA1 had a higher number of mutations than gRNA2 (Supplementary Table S2). 

To further ensure successful gene editing, two gRNAs were designed in the coding sequence for each gene target spaced between 40–250 bp apart. If both are successful in creating mutations, they can either create a deletion spanning the two sites or induce two smaller mutations at each target site. Likewise, if one gRNA unexpectantly fails or induces mutations at a lower rate, the second gRNA serves as a backup, providing a high overall chance of induced mutations knocking out the gene function. All combinations were observed in the present study in the edited progeny: deletions spanning both gRNA sites, small mutations at both gRNA sites, and small mutations at one of the gRNA sites (Figure 3 and Figure 4). Notably, most of the mutations observed in the current study were predicted to lead to frameshift mutations (such as the 1, 2, or 4 bp deletions or insertions). Moreover, in all cases where an in-frame mutation was expected at one gRNA site (i.e., four observed 3 bp deletions and one observed 6 bp deletion), the second gRNA site in the same plant had a predicted frameshift mutation (Figure 3 and Figure 4). Frameshift mutations are predicted to have a high chance of gene knockouts by leading to premature stop codons and truncated proteins. 

Another important component of a successful gene editing project is robust gRNA expression. Although both gRNAs can each be expressed with their own small nuclear RNA promoters, such as U3 or U6, greater levels of expression have previously been demonstrated by taking advantage of the endogenous tRNA processing system [44]. By inserting a synthetic polycistronic tRNA–gRNA (PTG) module into the plant, multiple gRNAs can be expressed at a high level and precisely excised in planta to produce functional gRNAs [44]. The current study deployed this system to express the two gRNAs for each target gene by ordering a synthetic DNA fragment specific for each gene target, which was then expressed with a U3 promoter, along with the Cas9 gene expressed with a rice ubiquitin promoter (Supplementary Figure S4). These vectors were tested using both *Agrobacterium*-mediated transformation and particle bombardment: calli were induced from mature seeds, transformed, regenerated, and grown until the desired visible phenotype was observed (Figure 1). As the *Agrobacterium*-mediated transformation and particle bombardment provided similar results, we chose to move forward with characterizing a subset of the *Agrobacterium*-transformed plants to avoid complications from multiple copies and/or fragmented insertions from bombardment. These results showed that stable integration by *Agrobacterium*-mediated transformation of a T-DNA containing the U3-PTG and UBI-Cas9 led to expression of both gRNAs and successful editing of each target gene (Supplementary Table S2).

The PDS protein plays a critical role in the carotenoid biosynthesis pathway and is involved in the synthesis of key photosynthetic pigments; notably, disruption of the *PDS* gene affects chloroplast development and leads to an albino phenotype [27]. Likewise, the YSA protein is important for the development of chloroplasts during early seedling development; disrupting the *YSA* gene causes an albino phenotype at the third-leaf stage; however, plants recover to a normal green by the six-leaf stage [38]. One objective of the current study was to compare the usefulness of knocking out the *PDS* and *YSA* genes to provide a visible phenotype when optimizing plant transformation and gene editing protocols in rice. Using CRISPR/Cas9 to edit the PDS gene produced both variegated and full albino plants (Supplementary Figure S1), while mutating the YSA gene produced a mix of green and more subtle variegated phenotypes (Supplementary Figure S2). Sanger sequencing was performed to confirm the mutations underlying the two gRNAs for each of the *PDS* and *YSA* target sites. For the *PDS* target, the sequence results largely matched the phenotypes—all of the albino seedlings had either a homogyous (i.e., two identical mutant alleles) or biallelic (i.e., two different mutant alleles) at one or both of the gRNA sites (Supplementary Table S2). Likewise, one of the green seedlings (PDS-4) had a heterozygous mutation at gRNA1 and was wild type of gRNA2, suggesting that the heterozygous mutation was not enough to cause an albino phenotype. At the same time, four other green PDS-edited seedlings (PDS-5, 6, 8, and 9) showed heterozygous mutations at gRNA1, but were homozygous or biallelic at gRNA2. A closer examination of the sequences from the five cloned PCR products for each transformed sample revealed that all four samples were heterozygous at gRNA1 (i.e., one wild type and one mutated allele) and that the homozygous or biallelic mutations at gRNA2 were largely 1 bp substitutions or 3 bp deletions, which presumably are not enough to disrupt the protein function (data not shown). On the other hand, mutations in the *YSA* gene did not lead to clear albino phenotypes. While all of the sequenced progenies had homozygous or biallelic mutations at both gRNA1 and gRNA2, only four plants showed either young white or variegated leaves (Supplementary Table S2). Although the published young seedling albino mutations had obvious albino phenotypes at the three-leaf stage, that was in the *indica* genetic background of Pei’ai64S [38]. It may be possible that the *tropical japonica* background (in this case, Presidio) has other related genes that compensate for the *YSA* mutations, leading to a reduced phenotypic effect. Therefore, the results of our study suggest that *PDS* is a more reliable phenotypic indicator than *YSA* for testing CRISPR-based knockouts in a *tropical japonica* genetic background.

## 4. Materials and Methods

### 4.1. Amplification of the PDS and YSA Genes in Nipponbare and Presidio

About 30–50 mg of the tips of rice leaves were collected and placed in 2.0 mL screw cap tubes (Cat #1420-9600, USA Scientific, Ocala, FL, USA) for DNA isolation. Genomic DNA extractions of rice leaf tissues were carried out following a modified CTAB protocol [45]. Briefly, leaf tissues were homogenized in 600 μL of CTAB buffer, 40 mg of polyvinylpyrrolidone (PVP-40) and 4 μL of 2-mercaptoethanol. Each sample was topped with 10 μL of RNase A (10 mg/mL), and then incubated at 60 °C for 15 min. Afterward, samples were extracted with chloroform: isoamyl alcohol (24:1), and genomic DNA was precipitated with 500 μL of isopropanol and resuspended in 100 μL of water.

To compare the similarity of both *PDS* and *YSA* between the Nipponbare and Presidio, primers were designed to the Nipponbare reference genome to amplify a portion of the *PDS* (Gramene ID: Os03g0184000) and *YSA* (Gramene ID: Os03g0597200). A portion of the *PDS* gene, which included exon 3 and exon 4, was amplified using the following primers: *OsPDS* forward primer (5’ CCATTACAGGTCGTGATTGCT) and *OsPDS* reverse primer (5’ ATCTATCAGTGCTGGCGGTAA). The following primers were used to amplify a part of the first exon of the *YSA* gene: *OsYSA* forward primer (5’ CCTGTGCATGCGCTCTCTTC) and *OsYSA* reverse primer (5’ AGGGGCACCAGGTGAAATTG). The PCR protocol for *PDS* is as follows: 98 °C for 30 s, followed by 30 cycles of 98 °C for 10 s, 60 °C for 30 s, and 72 °C for 30 s and a final extension of 72 °C for 5 min. The same PCR protocol was followed for *YSA*, with the exception that the annealing temperature was 58 °C. The fragments of the *PDS* and *YSA* genes were amplified in Nipponbare and Presidio cultivars using PhusionTM High-Fidelity DNA polymerase (Cat #F530L, ThermoFisher Scientific, Waltham, MA, USA) and viewed in a 1.2% agarose gel. The PCR products were extracted and purified using the QIAquick Gel Extraction Kit (Cat #28704, Qiagen, Germantown, MD, USA) and sequenced by Sanger sequencing (Texas A&M Laboraotry for Genome Technology, College Station, TX, USA). Conserved sequences between Presidio and Nipponbare were used for gRNA design. 

### 4.2. Design of gRNAs and Testing by In Vitro Digestion with the RNP Assay

Two gRNAs were designed for the coding regions of both *PDS* and *YSA*. All gRNAs were designed using the online CRISPRdirect design tool [46], and gRNAs with the NGG PAM sequence were selected. The *PDS* gene is located on chromosome 3 and has 14 exons. Two gRNAs targeting the third and fourth exon were designed (Supplementary Table S1). Coincidently, the gRNA targeting the fourth exon has been shown to work previously [47]. Both gRNAs are 204 bp apart from each other. The *YSA* gene, also located on chromosome 3, has one exon; therefore, both gRNAs were designed for this exon. The gRNAs targeting the *YSA* exon are 40 bp apart. 

Synthetic gRNAs targeting *PDS* and *YSA* were ordered from Synthego and their functionality was tested by in vitro digestion of target PCR amplicons using a ribonucleoprotein (RNP) assay. The PCR-amplified fragments of the *PDS* and *YSA* gene targets from wild-type Presidio were gel purified using a QIAquick gel extraction kit. The RNP complex was developed following a protocol from Integrated DNA Technologies (IDT) as follows: 11 μL of gRNAs with a concentration of 110 μM, 1.6 μL of 62 μM Cas9 protein from *Streptococcus pyogenes* (Cat #1081059, IDT, Coralville, IA, USA), and 87.4 μL of PBS buffer were combined and incubated at room temperature for 10 min. Then, 1 μL of RNP complex (1 μM) was mixed with 1 μL of 10x Cas9 nuclease reaction buffer (200 mM HEPES, 100 mM MgCl2, 5 mM DTT, and 167 mM KCl), 50 nM of DNA substrate, and 7.5 μL of nuclease-free water. Afterward, the mixture was incubated at 37 °C for 60 min, followed by the addition of 1 μL of 20 mg/mL proteinase K, which was then incubated at 56 °C for 10 min. Results were then visualized by electrophoresis on a 1.2% agarose gel. The cleavage efficiency was determined by densitometry using ImageJ 1.52v (https://imagej.nih.gov/ij/ accessed on 20 April 2020). The control contained everything but the gRNAs, which resulted in an uncut PCR amplicon of either *PDS* or *YSA*, and the controls were compared to the products that were cut by the gRNA.

### 4.3. Construction of the tRNA–gRNA Vector and Agrobacterium Cloning

To obtain efficient expression of both gRNAs targeting the *PDS* and *YSA* genes, we used an endogenous tRNA-processing system, which has previously been shown to work well in rice [44]. BsaI restriction sites flanking both ends of the tRNA–gRNA construct were used for the ligation to the pRGEB32 vector (Addgene plasmid #63142; http://www.addgene.org/63142/ accessed on 20 April 2020). The tRNA–gRNA construct was synthesized by GenScript and cloned into a pUC57 vector by GenScript. This vector was then digested with BsaI to extract the tRNA–gRNA construct and ligated to the pRGEB32 vector. The end result was the pRGEB32 binary vector with either the *PDS* tRNA–gRNA or *YSA* tRNA–gRNA construct (Supplementary Figure S4), which was then transformed into 5-alpha *E. coli* competent cells (Cat #C2988J, NEB, Ipswich, MA, USA). The *E. coli* transformation protocol was performed as suggested by NEB. The tRNA–gRNA and pRGEB32 plasmid were isolated from *E. coli* using the QIAprep Spin Miniprep Kit (Cat #27104, Qiagen, Germantown, MD, USA), validated by both restriction digestion and Sanger sequencing. Validated plasmids were then transformed into *Agrobacterium tumefaciens* EHA105 cells.

EHA105 transformations with the tRNA–gRNA fragment in pRGEB32 were carried out using the freeze and thaw method [48]. Briefly, 50 μL of EHA105 *Agrobacterium* cells were thawed on ice, and 1 μL (100–1000 ng) of plasmid DNA was added. The tube was gently flicked to mix, and the mixture was kept on ice for 2 min. The mixture was then transferred into liquid nitrogen for 5 min and then incubated in a 37 °C water bath for 5 min. After the incubation, 1 mL of liquid LB media was added to the mixture and incubated for 4 h on a shaking incubator at 28 °C. The tubes were then placed in a centrifuge for 2 min at 6000 rpm to pellet the cells. The excess supernatant was removed and left with 50 μL of liquid LB media. All 50 μL were then suspended on Petri dishes containing solid LB media with kanamycin antibiotics and incubated for 2 days at 28 °C. Colonies were picked and placed in 5 mL of liquid LB media on a shaking incubator at 28 °C for three days to allow the cells to grow. Approximately 4 mL of the plasmid was isolated for validation by restriction digestion and Sanger sequencing. The other 1 mL was stored to be used for the plant transformations. 

### 4.4. Agrobacterium-Mediated Transformation

The tissue culture transformation approach using the callus method for mature seeds was adapted from a previously published protocol [9] with some modifications. *Agrobacterium tumefaciens* EHA105 was cultured on an AB plate that contains appropriate antibiotics in the dark at 28 °C for 3 days. The bacteria were then collected with a loop, suspended in 1 mL of AAM medium at a final density OD600 = 0.3. Well-maintained mature Presidio seeds were dehusked and collected in a 50 mL sterile tube. Next, the seeds were surface sterilized in 20 mL of 70% ethanol for 10 sonds, followed by 25 mL of 2% sodium hypochlorite that contains one drop of Tween-20 under stirring for 30 min. The seeds were then washed in sterile water three to five times, placed them on 2N6 callus induction medium and incubated at 30 °C in the dark for 7 days. Scutella from cultured seeds were then cut out, transferred to fresh 2NBK medium with the scutella facing up, and incubated at 30 °C in the dark for 21 days for calli formation. The actively proliferating calli were cut/isolated into small pieces of 0.5–1 mm in diameter using a scalpel and were transferred with forceps to the 2N6 medium. Next, the plate was sealed with surgical tape, and 200–250 calli can be placed on a single plate. Following this, the plates were incubated under continuous illumination (5000 lux) at 32 °C for 3 days. 

The new actively proliferating calli were soaked in 5 mL sterile water in a 90-mm Petri dish. After that, the calli were separated and the water was removed. A total of 1.0 mL of bacterial suspension was added and mixed with the calli by pipetting; the mixture was then incubated for 2 min at 25 °C. The mixing by pipetting was repeated again and the bacterial suspension was then removed. Following this, approximately 150 calli were transferred with forceps to a co-cultivation medium, and the plate was sealed with Parafilm and incubated in the dark at 25 °C for 1 day. After co-cultivation, the calli were transferred to selective medium 2NBKCH40,30 to 60 calli can be placed on a single plate. Next, the plates were incubated under dark conditions at 32 °C for 2 weeks. Subsequently, the proliferated, yellowish white calli were transferred to the second selective medium NBKCH40 (modified) and the plate was sealed with surgical tape. The plates were then incubated under dark conditions at 32 °C for 7 days. Again, the proliferated calli were transferred to the regeneration medium NBKRH40, and the plate was sealed with surgical tape. The plates were then incubated under continuous illumination (5000 lux) at 32 °C. Thereafter, the subculture was performed into fresh media every three weeks until plant regeneration (40–60 days). Regenerated plantlets advanced to rooting induction media NBKFH40 under continuous illumination at 32 °C.

### 4.5. Particle Bombardment-Mediated Transformation

Preparation of gold particle stocks was performed as follows: a total of 20 mg of 0.6 μm gold microcarriers (Cat #165-2262, Bio-Rad, Hercules, CA, USA) were weighed in a sterile 1.5 mL Eppendorf tube followed by the addition of 1mL of 100% ethanol. The mixture containing gold and ethanol underwent water bath sonication (Cat #CPX-952-218R, Branson, Brookfield, CT, USA) for 2 min and was then microcentrifuged for 3 s at top speed. The supernatant was removed, and the ethanol wash was repeated once more. The wash with ethanol was performed twice. Afterward, 1 mL of sterile distilled water was added, and water bath was sonicated for 2 min. The gold microcarriers were then centrifuged for 3 s at top speed. The wash with sterile distilled water was repeated once more. The washes with distilled water were performed twice. Thereafter, the gold microcarriers were resuspended in 1 mL of sterile distilled water. The resuspension was aliquoted into 50 μL amongst 1.5 mL Eppendorf tubes and stored at −20°C for future use. 

### 4.6. Coating the Gold Microcarriers with Plasmid DNA

An aliquot of the gold microcarriers was defrosted and water-bath sonicated for 1 min. Plasmid DNA isolated with the Plasmid Maxi Kit (Cat #12162, Qiagen, Germantown, MD, USA) was used for particle bombardment in order to obtain a high concentration, which decreases the volume used during the experiment. Our experiments seemed to work best when the volume of DNA was 5 μL or less. At least 1 mg/mL of plasmid DNA was added to the gold microcarriers, and the Eppendorf tube was vortexed for 1 min. After mixing the components together, 50 μL of 2.5 M calcium chloride and 20 μL of 0.1 M spermidine was added onto the inside of the cap of the Eppendorf tube. The calcium chloride and spermidine were mixed together on the cap by pipetting up and down 2–3 times. The cap of the Eppendorf tube was closed, and the tube was tapped down to let all the components mix together at the bottom of the Eppendorf tube. The mixture was microcentrifuged for 5 s at top speed, and the supernatant was removed. The pellet was resuspended in 150 μL of 100% ethanol and vortexed for 1 min. The components were microcentrifuged for 5 s at top speed, and the supernatant was removed. The pellet was then resuspended in 40 μL of 100%.

### 4.7. Preparation of Plasmid DNA Delivery

The macrocarrier holders (Cat #165-2322, Bio-Rad, Hercules, CA, USA) and stopping screens (Cat #165-2336, Bio-Rad, Hercules, CA, USA) were sterilized by autoclave. The macrocarriers (Cat #165-2335, Bio-Rad, Hercules, CA, USA) and 900 psi rupture disks (Cat #165-2328, Bio-Rad, Hercules, CA, USA) were sterilized with 100% ethanol on the day of use. The Biolistic® PDS-1000/He particle delivery system (Cat #165-2257, Bio-Rad, Hercules, CA, USA) was cleaned and sterilized with 75% ethanol before and after use. Once cleaned with 100% ethanol, macrocarriers and rupture disks are placed in sterile Petri dishes and allowed to air dry. Once airdried, the macrocarriers are loaded on the macrocarrier holders. The gold-plasmid complex in the 100% ethanol solution was slowly loaded onto the center of the macrocarrier, which is within the macrocarrier holder. The gold-plasmid DNA complex was then allowed to air dry on the macrocarriers for 5–10 min. 

Once the gold-plasmid DNA has dried, the macrocarrier holder is loaded onto the delivery system. The set-up for the Biolistic® PDS-1000/He particle delivery system was performed following the provided manual. The settings used for the Biolistic® PDS-1000/He particle delivery system were as follows: 2.5 cm distance gap between the rupture disk and macrocarrier, 9 cm target distance between the stopping screen and the target plate, 0.8 cm distance between the macrocarrier and the stopping screen, 28–29” Hg vacuum, 5.0 vacuum flow rate, and 4.5 vacuum vent rate. Presidio rice calli on osmotic media where bombarded, and the plates were removed from the delivery system and wrapped with 3M micropore tape. The osmotic media was prepared as follows: 4.4 g of Murashige and Skoog (MS) media (Cat #M5524, Sigma-Aldrich, St. Louis, MO, USA), 5 mL of 2,4-dichlorophenoxyacetic acid (1mg/mL) (Cat #D72724, Sigma-Aldrich, St. Louis, MO, USA), 72.867g of mannitol (Cat #M1902, Sigma-Aldrich, St. Louis, MO, USA), and 3.2 g of phytagel (Cat #P8169, Sigma-Aldrich, St. Louis, MO, USA), and the pH was adjusted to 5.8 (Liang et al. 2018). The plates were placed in the dark at 25 °C overnight. After the overnight incubation in the dark, plates were kept under the same conditions as calli treated with *Agrobacterium*.

### 4.8. Validation of Edited Plants

Leaf samples from the regenerated plants, transformed with either a *PDS* tRNA–gRNA or *YSA* tRNA–gRNA plasmid, were collected. Genomic DNA was extracted as described above, and the regions of *PDS* and *YSA* targeted by the sgRNAs were amplified by PCR. The purified PCR samples were gel purified and ligated to pCR–Blunt vector (Cat #K270020, ThermoFisher Scientific, Waltham, MA, USA). The ligated vector and PCR product were then transformed into DH5-alpa competent *E. coli* cells. For each sample, five colonies were randomly selected, and DNA plasmids were then isolated using QIAprep Spin Miniprep Kit and were sent for Sanger sequencing at the Laboratory for Genome Technology at Texas A&M University using an ABI 3130 Genetic Analyzer. Sequencing data were then analyzed using the MAFFT Multiple Sequence Alignment Software Version 7 from Benchling (https://www.benchling.com/ accessed on 20 April 2020).

## 5. Conclusions

The current study has optimized an *Agrobacterium*-mediated transformation protocol using callus induction from mature seeds of the high-yielding U.S. *tropical japonica* cultivar Presidio. Gene editing efficiency was tested by evaluating knockout mutations in the *phytoene desaturase* (*PDS*) gene, which provided a clear visible phenotype at the seedling stage for successful knockouts. Sanger sequencing of edited progeny showed a number of insertions, deletions, and substitutions at the gRNA target sites. This optimized protocol provides an efficient mature-seed transformation protocol for a high-yielding *tropical japonica* variety and paves the way for rapid and efficient CRISPR-based gene editing in the background of popular, high yielding, long-grain rice cultivars for rice production in the U.S. and South America.

## Figures and Tables

**Table 1:** Modifications of the optimized steps in comparison with the previously published protocol.

	Callus Induction	Co-Cultivation	1^st^ Selection	2^nd^ Selection	Regeneration
Hiei and Komari [9]	10 days/dark	3 days/dark	14 days/light	5 days/light	14 days/0.5mgl^−1^ kinetin light
Optimized Protocol	21 days/dark	1 day/dark	14 days/dark	7 days/dark	40 days/3mgl^−1^ kinetin light

**Table 2:** Numbers of transformed explants, regenerated plants, and albino plants regenerated.

Experiment Name	Vector Treatment	No. Explants Treated	No. Plants Regenerated	No. Albino Plants Regenerated
T.9.08-Agro	pRGEB32-PDS	88	31	16
T.9.08-Agro	pRGEB32-YSA	59	18	-
T.9.09-Agro	pRGEB32-PDS	123	18	14
T.9.09-Agro	pRGEB32-YSA	262	75	-
Agro ^1^	empty pRGEB32 control	100	44	8
BB#1	pRGE32-PDS	148	33	14
BB#1	pRGE32-YSA	195	154	-
BB#2	pRGE32-PDS	161	24	20
BB#2	pRGE32-YSA	144	36	-
BB^2^	empty pRGEB32 control	62	9	

^1^ Agro: *Agrobacterium*-mediated transformation; ^2^ BB: bombardment (biolistic transformation).

## Data Availability

All data is available in the Supplementary Materials.

## Supplementary Materials

The following are available online at https://www.mdpi.com/article/10.3390/ijms222010909/s1, Table S1: Sequences of gRNAs and PAMs targeting the PDS and YSA genes, Table S2: Phenotypes of edited progeny and mutations at targeted loci, Figure S1: Phenotypes of *PDS* edited progeny, Figure S2: Phenotypes of *YSA* edited progeny, Figure S3: Sanger sequence data from five cloned PCR products for each of four representative edited progeny for the *PDS* gene target, Figure S4: Plasmid maps of the pRGEB32 binary vector for the (a) *PDS* and (b) *YSA* gene targets.
