# Supplementary material for: Optimizing Agrobacterium-Mediated Transformation and CRISPR-Cas9 Gene Editing in the tropical japonica Rice Variety Presidio
Source: Int J Mol Sci. 2021 Oct 9;22(20):10909. doi: 10.3390/ijms222010909 (PMC8535416; doi:10.3390/ijms222010909)
Supplement: Supplementary file 1 [file ijms-22-10909-s001.zip › ijms-1389143-supplementary.pdf]

Supplementary Materials

Supplementary Table S1. Sequences of gRNAs and PAMs targeting the *PDS* and *YSA* genes

Target	gRNA + **PAM**	Target region
*Os*PDS sgRNA1	5’ GCAAAATATCTGGCAGATGC**TGG**	Exon 3
*Os*PDS sgRNA2	5’ GGAAGGATGAAGATGGAGAT**TGG**	Exon 4
*Os*YSA sgRNA1	5’ **CCC**CGCGTTTGCGCCGCCCCTCG	Exon 1
*Os*YSA sgRNA2	5’ **CCG**CTTCGGCCGAGGTGGCGCGC	Exon 1

(a)

Gene	Phenotype	gRNA1	gRNA2
PDS 1	White	Homozygous	Biallelic
PDS 2	White	Homozygous	Homozygous
PDS 3	White	Homozygous	Homozygous
PDS 4	Green	Heterozygous	Wild-type
PDS 5	Green	Heterozygous	Biallelic
PDS 6	Green	Heterozygous	Homozygous
PDS 7	Variegated	Heterozygous	Biallelic
PDS 8	Green	Heterozygous	Biallelic
PDS 9	Green	Heterozygous	Biallelic
PDS 10	White	Homozygous	Biallelic
PDS 11	White	Heterozygous	Biallelic
PDS 12	White	Biallelic	Biallelic
PDS 27 high	White	Homozygous	Homozygous
PDS 27 low	White	Homozygous	Homozygous
PDS 28 high	White	Homozygous	Homozygous
PDS 28 low	White	Biallelic	Homozygous
PDS 29 high	White	Homozygous	Biallelic
PDS 29 low	White	Homozygous	Homozygous
PDS 30	White	Homozygous	Homozygous
PDS 31	White	Homozygous	Homozygous
PDS 32	White	Homozygous	Biallelic

(b)

Gene	Phenotype	gRNA1	gRNA2
YSA 17	Green	Homozygous	Homozygous
YSA 19	Green	Homozygous	Homozygous
YSA 20	Green	Biallelic	Homozygous
YSA 21	Green	Homozygous	Homozygous
YSA 22	Green	Homozygous	Homozygous
YSA 23	Green	Biallelic	Biallelic
YSA 24	Green	Homozygous	Homozygous
YSA 25	Green	Homozygous	Homozygous
YSA 26	Green	Homozygous	Homozygous
YSA 35.1	Green leaf part	Homozygous	Homozygous
YSA 36	Green	Biallelic	Homozygous
YSA 37	Young white leaf	Homozygous	Biallelic
YSA 38	Young white leaf	Biallelic	Homozygous
YSA 39	Green	Biallelic	Homozygous
YSA 40	Variegated	Homozygous	Homozygous
YSA 41	Variegated	Homozygous	Homozygous

Supplementary Table S2. Phenotypes of edited progeny and mutations at targeted loci. Visible phenotypes (white, green, or variegated leaves) and mutations detected at each gRNA site for (a) *PDS* and (b) *YSA*, including homozygous monoallelic, biallelic, heterozygous, and wild type.

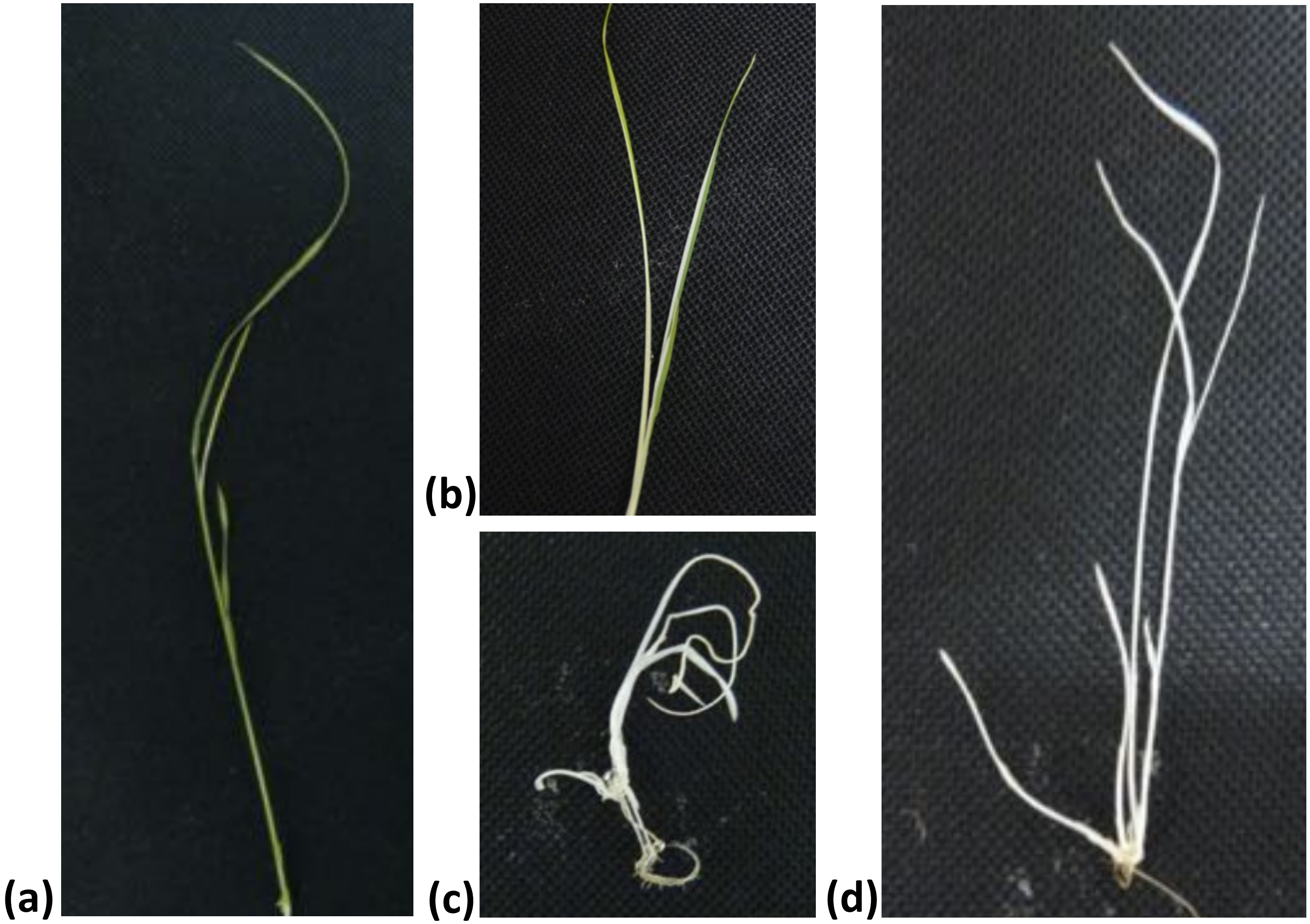

Supplementary Figure S1. Phenotypes of *PDS* edited progeny. The sgRNAs targeting the *PDS* gene induced several different phenotypes from the gene knockouts: (a) empty vector controls appeared as green wild type plants; while various mutations appeared as (b) variegated plants, (c) dwarf albino plant, and (d) normal-size albino plant.

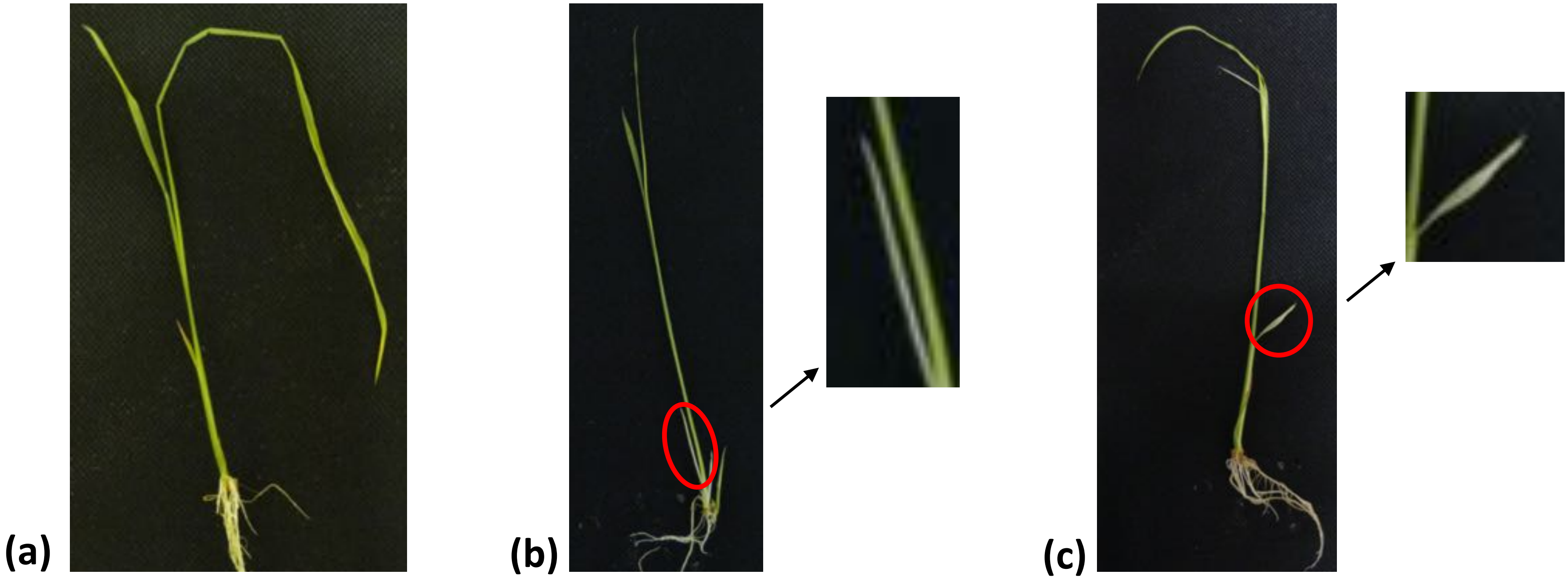

Supplementary Figure S2. Phenotypes of *YSA* edited progeny. The sgRNAs targeting the *YSA* gene induced more subtle phenotypes from the gene knockouts: (a) empty vector controls appeared as green wild type plants; while mutations appeared as (b) variegated plants with green and albino sectors and (c) variegated plants with albino leaf tips.

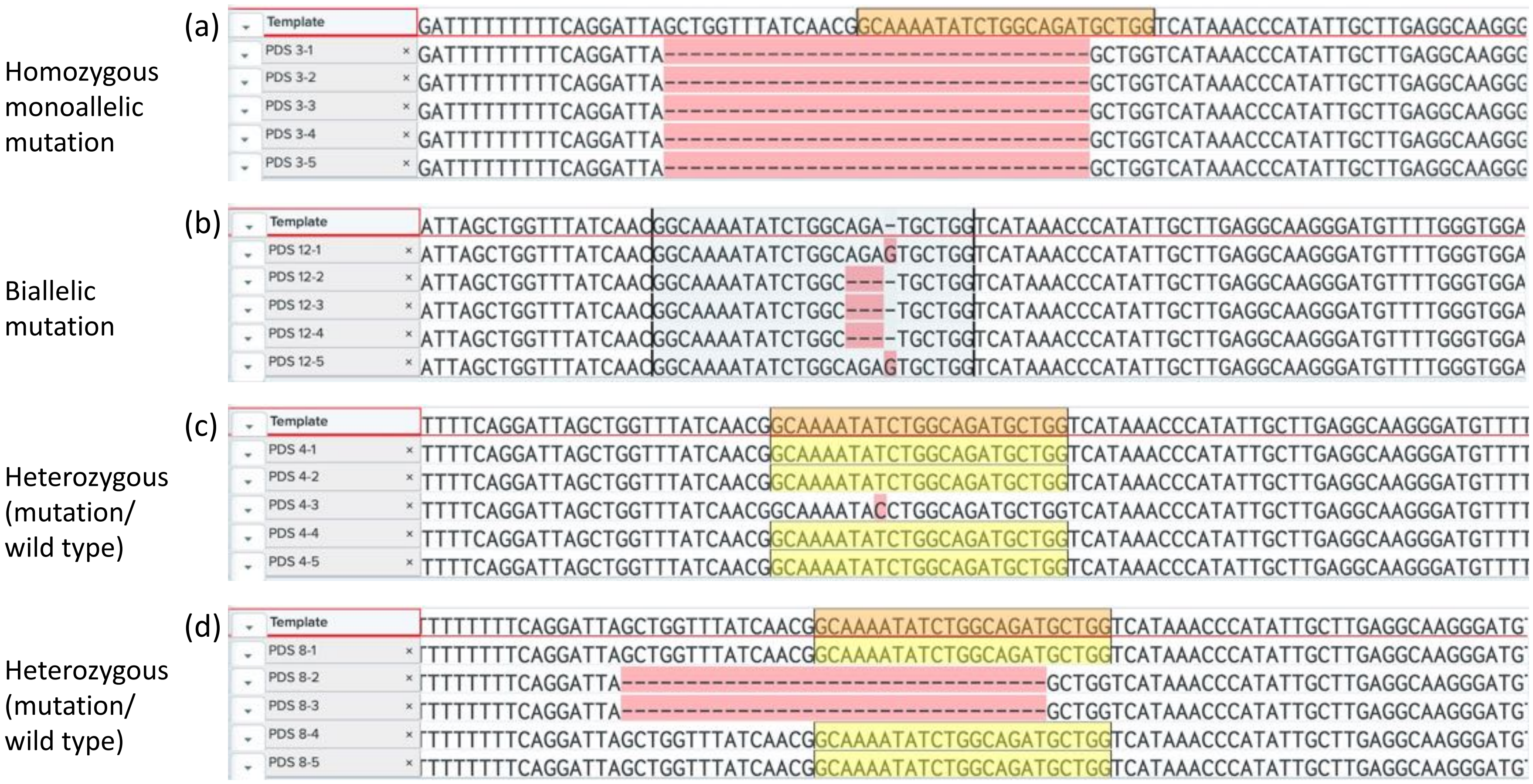

Supplementary Figure S3. Sanger sequence data from 5 cloned PCR products for each of four representative edited progeny for the PDS gene target. Sequence analysis revealed three types of alleles: (a) homozygous monoallelic mutation (in this case a 33 bp deletion); (b) biallelic mutation (3 bp deletion and a 1 bp insertion); (c) heterozygous (1 bp substitution and wild type allele); and (d) heterozygous (33 bp deletion and wild type allele).

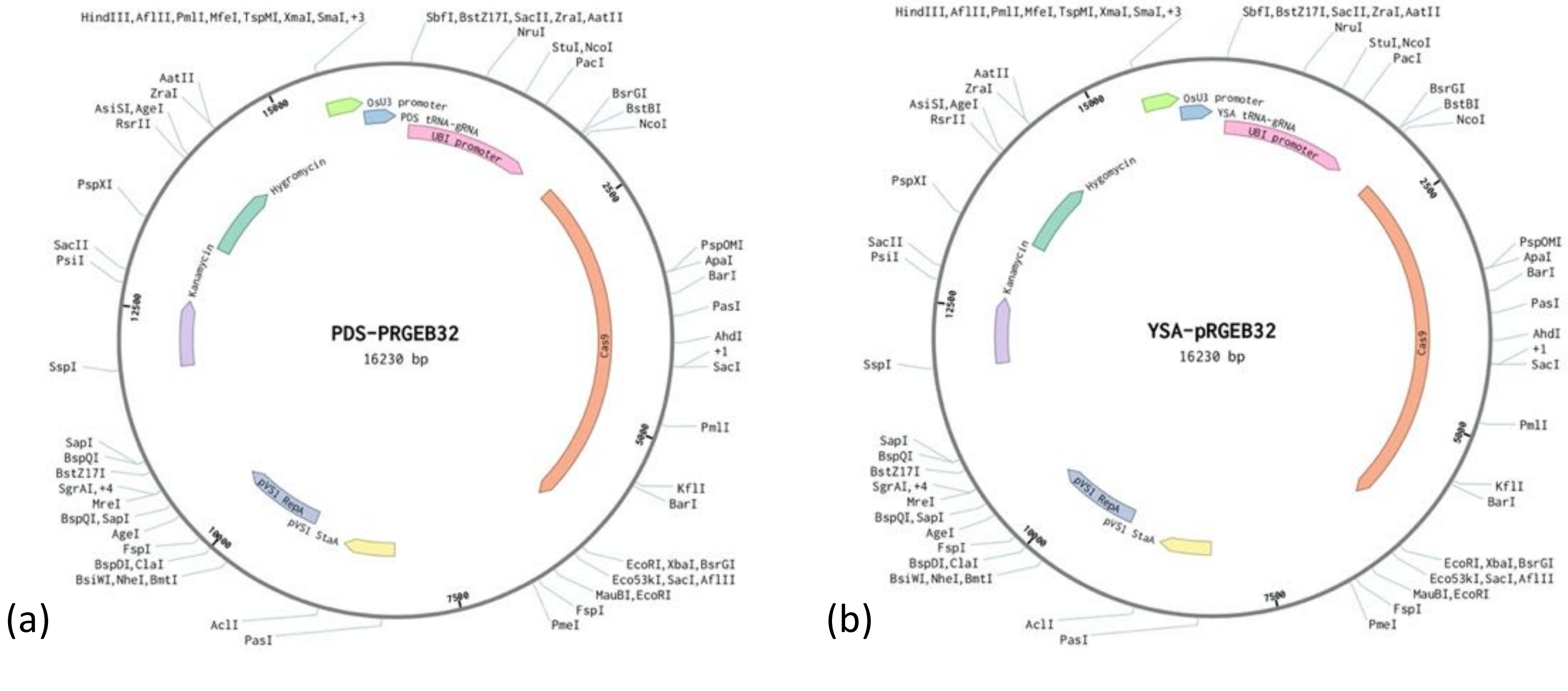

Supplementary Figure S4. Plasmid maps of the pRGEB32 binary vector for the (a) *PDS* and (b) *YSA* gene targets. A synthetic DNA fragment containing the polycistronic tRNA-gRNA module was inserted behind the U3 promoter in the pRGEB32 binary vector (Addgene Plasmid #63142), which also has selectable markers and Cas9 expressed from a ubiquitin promoter.
